# Reform of agricultural land property rights system and grain production resilience: Empirical evidence based on China’s “Three Rights Separation” reform

**DOI:** 10.1371/journal.pone.0319387

**Published:** 2025-03-21

**Authors:** Zeng Wei, Jiquan Peng, Yan Zhao, Xijian Li, Cheng Wang

**Affiliations:** 1 School of Business Administration, Zhongnan University of Economics and Law, Wuhan, China; 2 School of Economics, Jiangxi University of Finance and Economics, Nanchang, China; 3 School of Tourism Management, Wuhan Business University, Wuhan, China; 4 College of Economics and Management, Hubei University of Arts and Science, Xiangyang, China; Southwestern University of Finance and Economics, CHINA

## Abstract

The latest reform in China’s agricultural land rights system has implemented the “Three Rights Separation” reform, distinguishing between ownership rights, contract rights, and management rights of rural land. This reform is a significant step taken by the Chinese government to ensure a rational allocation of agricultural land resources, contributing greatly to enhancing food production resilience and promoting food security. This paper analyzes panel data from 30 Chinese provinces from 2005 to 2021 to assess the resilience levels in grain production. It utilizes a multi-period Difference-in-Differences model to examine the effects of the separation of three rights of agricultural land on this resilience, including their mechanisms of action. The findings reveal that(1)although the resilience of grain production in China has progressively improved over the study period, it remains low, indicating substantial potential for enhancement;(2)The separation of three rights of agricultural land significantly boosts the resilience of grain production, a conclusion corroborated by robustness tests;(3)The analysis shows that the policy promotes resilience by facilitating land transfer and boosting investments in agricultural productivity;(4)Heterogeneity analysis indicates that the policy’s impact is more pronounced in major grain-producing areas and central regions, with stronger effects observed in northern wheat-growing areas compared to southern rice-growing regions.

## 1 Introduction

The food security is a major strategic issue concerning national security and the sustainable development of socioeconomic stability. In 2023, China’s grain production reached a historic high of 695 billion kilograms, utilizing 9% of the world’s arable land to feed approximately 22% of the global population [1]. However, it is also important to recognize the numerous risks and challenges currently faced by China in grain production, including the increased natural risks from extreme climate events [2], the external shocks from international grain price fluctuations [3], and the “non-grain” conversion of arable land due to low grain-farming profitability [4]. The inherent vulnerability of the grain production system limits its capacity to withstand and mitigate external risks. Should these risks profoundly disrupt the grain production system, the economic and social security of the nation would face significant impacts.Resilience in grain production demonstrates the ability to respond to and adapt to shocks brought about by natural disasters, market fluctuations, and other uncertain factors. Enhancing the resilience of grain production can mitigate the negative impacts of yield volatility, strengthen the stability and sustainability of grain production, reduce the prevalence of hunger and poverty, promote rural economic development, and maintain social stability.In this context, constructing a grain production system with higher resilience is crucial for ensuring food security under diverse and complex conditions. Therefore, enhancing the resilience of grain production and developing a more sustainable long-term food security mechanism are critical issues that China needs to address now and in the future.

Resilience was initially applied in the field of physics and has gradually been incorporated into ecology, psychology, economics, and other disciplines. Scholars have conducted extensive research on the essence, evaluation systems, measurement methods, and enhancement strategies of resilience from various disciplinary perspectives and research viewpoints. Tendall et al. developed a conceptual framework for resilience in food systems, defining it as the capacity of food systems to withstand shocks [5]. This capacity is potentially constituted by socioeconomic, biophysical, and production diversity aspects [6], and is manifested in the food system’s flexible response to risk shocks through absorption, reaction, adaptation, learning, and ensuring sustainability [7]. Meanwhile, a minority of scholars have measured the development level of grain production resilience by constructing an evaluation index system [8,9] and conducted empirical analysis on the impact of climate change on grain production resilience [10]. Among the institutional factors affecting food production, the system of agricultural land property rights is paramount.

The “Three Rights Separation” reform distinguishes ownership, contract, and management rights in land contracted by households. Building on the household responsibility system which initially separated ownership and operational rights this reform further differentiates contract rights from management rights. Widely recognized as a pivotal innovation in land rights since the household responsibility system, the reform has reignited momentum in agricultural and rural policy development in China. China’s “three rights separation” reform began in 2014 when the government issued the “Guiding Opinions on the Orderly Transfer of Rural Land Management Rights to Develop Appropriate Scale Agricultural Operations.” This policy emphasized collective ownership of rural land and encouraged the separation of ownership, contract, and management rights to facilitate the structured transfer of land management rights. Subsequently, the “Comprehensive Implementation Plan for Deepening Rural Reform” (2015) advocated for the stabilization of farmers’ contract rights and liberalization of management rights through the three rights separation. The formal establishment of land management rights in the revised “Rural Land Contract Law” (2019) transitioned the reform from policy to legislation, solidifying the legal framework for “three rights separation.” This latest phase of land rights reform has spurred consider-able academic debate, with research examining both its theoretical implications and practical applications. Theoretically, scholars have delved into the institutional significance and structure of the “three rights separation” framework [11], offering reflections on its practical applications and potential developmental trajectories [12,13]. On a practical level, research has investigated the impact of “three rights separation” on various dimensions of agricultural productivity [14], including the promotion of green agricultural practices [15], modern agricultural development efficiency [16], arable land output efficiency [17], agricultural non-point source pollution [18], farmer incomes [19,20], and endowment effects [21].

In summary, existing literature on agricultural land “three rights separation” and food production resilience provides a robust theoretical foundation for this study, yet reveals several gaps: (1) Research on factors influencing food production resilience remains relatively sparse, with limited focus on how land rights reform policies affect this resilience; (2) the mechanisms by which agricultural land “three rights separation” may bolster food production resilience require further examination; and (3) despite substantial policy discussions on agricultural land “three rights separation,” empirical analyses remain underdeveloped. The potential contributions of this paper are three fold: (1) advancing the research on methods for measuring food production resilience; (2) empirically assessing the policy impact of agricultural land “three rights separation” on enhancing food production resilience; and (3) offering an indepth analysis of the mechanisms by which agricultural land “three rights separation” promotes resilience in food production.

## 2 Theoretical analysis and research hypotheses

### 2.1 Land transfer mechanism

A well-defined property rights system is fundamental for enabling the fluid movement of land. Through the issuance of legally binding certification documents, the reform strengthens the stability and security of farmers’ land contracting rights, thereby increasing the stability, security, and exclusivity of agricultural land property rights [22]. This legal assurance reduces farmers’ concerns about potential risks associated with long-term rentals, boosting their willingness to transfer agricultural land [23]. Building on the existing separation of ownership and contracting rights, the separation of three rights reform introduces an additional division between contracting and management rights, further expanding the functional scope of agricultural land property rights. At the policy level, the reform encourages the transfer of management rights, raising expectations for returns on these transfers and promoting the outward movement of low-return or underutilized farmland. In parallel, rural land management right transfer markets have been established across various regions, along with strengthened oversight to facilitate orderly transactions. These developments contribute to a more market-driven transfer of agricultural land across regions.

Land transfer facilitates the intensification of agricultural land management. It allows agricultural land resources, which are less efficient in grain production and where contractors have a strong desire to exit grain planting and expect to earn income from land transfers, to be transferred to contractors with higher grain production efficiencies, achieving more intensive land management. Intensified management of land aids in the scientific combination and intensive use of grain production factors, significantly increasing per unit grain yield, and also allows the cost of agricultural machinery and land improvements to be spread over a larger area. Land transfer is a necessary prerequisite for moderate-scale operations [24], which are a crucial pathway to enhancing the efficiency of grain production [25]. On one hand, the costs of production and market transactions gradually decrease at higher scales of grain crop production, and compared to cash crops, grain crops exhibit stronger economies of scale, resulting in higher returns from grain cultivation than from non-grain crops [26], thereby boosting farmers’ enthusiasm for continuous grain production. Therefore, land transfer can lead to a ’grain-oriented’ trend in land production [27]. On the other hand, scaled operations can mitigate the impacts of aging rural labor forces, such as abandoned or extensively managed farmland, and increase the use of modern grain production technologies and management practices like improved seeds, mechanization, insurance, meteorological services, and scientifically balanced fertilizers and pesticides, enhancing the resilience of grain production to risks.

### 2.2 Agricultural productive investment mechanism

When agricultural land rights are unstable, farmers perceive a heightened risk of future land infringement, reducing their motivation to invest in land over the medium to long term. By formalizing the legal status of farmers’ land contracting rights, the reform enhances their sense of security and ownership, thereby increasing their willingness to make productive investments. This reform promotes the consolidation of management rights among farmers with higher production efficiency and a stronger commitment to agricultural production, while securing stable contracting rights. For farmers acquiring land management rights, the clarity in defining the transferred land area and the long-term stability of these rights lower transaction costs and mitigate contract breach risks, fostering investment incentives driven by economies of scale. Compared to small-scale farmers, larger agricultural operators benefit from easier access to land management right mortgages, which expands their investment capacity in agricultural production [28]. Furthermore, these larger-scale operators are more inclined and able to adopt green production technologies, thereby enhancing both their engagement with and investment in sustainable agricultural practices.

Investment in agricultural machinery can reduce labor costs [29], increase the possibility of multiple cropping [30], and enhance the ecological coordination and foundational capabilities of grain production. The input of agricultural machinery also strengthens the resilience and regenerative capacity of grain production through the “technology introduction effect.” On one hand, it can enhance the technological coverage across various stages of grain production. Technological innovations in machinery can enhance the resilience and regenerative abilities of grain production; for example, during the seeding phase, mechanical seeding not only improves seeding efficiency, but also allows for precise monitoring of soil moisture, nutrient status, and crop growth [31], enhancing the capacity of grain production to withstand natural risks. During the harvesting phase, mechanical harvesting efficiency significantly surpasses manual harvesting, and can better avoid harvesting losses. On the other hand, investment in agricultural mechanization can stimulate advances in grain production technology.

Based on the above analysis, this paper proposes the following research hypotheses:

H1: The Reform of the Separation of Three Rights of Agricultural Land Enhances Resilience in Grain Production.

H2: Enhancing Grain Production Resilience Through Agricultural Land Transfer in the Separation of Three Rights Reform.

H3: Enhancing Grain Production Resilience Through Agricultural Productive Investment Under the Separation of Three Rights Reform.

## 3 Empirical research design

### 3.1 Model setup

#### 3.1.1 Baseline regression model

The DID model,as a tool for policy evaluation, has gained increasing favor among scholars in recent years.This study employs a DID model,leveraging the agricultural land “three rights separation” reform as a quasi-natural experiment to analyze the effects of land rights reform on food production resilience.Although the “three rights separation” reform was formally introduced by the Chinese government in 2014, but its implementation timeline varied across local governments.However,traditional DID models,which typically examine policies with only a single shock point,cannot accurately estimate the effects of policies that have multiple shock points. Therefore,this paper adopts a multi-period DID model to assess the policy effects of the separation of three rights of agricultural land. The empirical model constructed in this paper is as follows:


$$\begin{array}{ccc} {Resil}_{it}=\beta_{0}+\beta_{1}{DID}_{it}+\beta_{2}{Control}_{it}+\delta_{i}+\theta_{t}+\varepsilon_{it} & & \end{array}$$ (1)

In Formula (1): ${Resil}_{it}$ is the dependent variable,representing the resilience of grain production in province i during period t; ${Resil}_{it}$ is the explanatory variable,coded as 1 for years after the initiation of land titling and 0 otherwise; ${DID}_{it}$ includes control variables such as regional economic development level,urbanization rate,rural human capital, farmer income,agricultural industry concentration, regional industrial structure, and agricultural insurance density. Additionally, $\beta_{0}$ represents the constant term; $\beta_{1}$ indicates the policy effect; $\beta_{2}$ are the coefficients for the control variables; $\delta_{i}$ represents fixed effects for provinces; $\theta_{t}$ represents fixed effects for years; $\varepsilon_{it}$ is the error term.

#### 3.1.2 Parallel trends test model

The validity of the multi-period DID model estimation is contingent on meeting the parallel trends test. This paper uses the event study approach to examine the trend changes in grain production resilience before and after the separation of three rights of agricultural land, with the specific model being:


$$\begin{array}{ccc} {Resil}_{it}=\beta_{0}+\overset{2021}{\underset{2005}{\sum }}\beta_{t}{DID}_{it}+\beta_{2}{Control}_{it}+\delta_{i}+\theta_{t}+\varepsilon_{it} & & \end{array}$$ (2)

In Formula (2), ${DID}_{it}$ represents a set of dummy variables,assigned a value of 1 if land titling was implemented in province i during year t,and 0 otherwise; $\beta_{0}$,$\beta_{t}$,$\beta_{2}$ are the coefficients to be estimated; $\delta_{i}$,$\theta_{t}$, $\varepsilon_{it}$ retain the same meanings as in Formula (1).

### 3.2 Variable selection

#### 3.2.1 Dependent variable

Based on previous studies [8,32], this paper defines grain production resilience as the capacity of the grain production system to maintain or swiftly recover its production functions in the face of various unforeseen shocks, while undergoing fundamental transformations to ensure the stability of grain supply and enhance its capacity for sustainable development.A resilience evaluation index system for grain production has been constructed,encompassing three dimensions resilience, recovery, and regeneration including six secondary indicators and twenty-one tertiary indicators,as detailed in Table 1. Given the suitability of the entropy method for evaluating different research subjects across multiple periods,this method will also be used to determine the weights of the indicators. Based on this, a comprehensive index of the resilience development level of grain production in various provinces from 2005 to 2021 will be calculated. Additionally, this paper employs the coefficient stripping method to isolate grain production inputs from broader agricultural inputs for some indicators.

**Table 1:** Evaluation index system for grain production resilience.

Primary Indicator	Secondary Indicator	Tertiary Indicator	Variable Code	Indicator Attribute
Resistance capacity	Foundational security of food production	Effective Irrigated Area (thousand hectares)	A1	+
		Grain Sown Area (thousand hectares)	A2	+
		Number of Workers in the Primary Industry (ten thousand people)	A3	+
	Stability of Grain Production Capacity	Grain Output Fluctuation Coefficient (%)	A5	-
		Per Capita Grain Possession (kilograms per person)	A6	+
		Grain Yield per Unit Sown Area (kilograms per hectare)	A7	+
Restorative capacity	Ecological Coordination of Grain Production	Pesticide Use per Unit Grain Sown Area (kilograms per hectare)	B1	-
		Agricultural Diesel Use per Unit Grain Sown Area (kilograms per hectare)	B2	-
		Fertilizer Use per Unit Grain Sown Area (kilograms per hectare)	B3	-
		Agricultural Plastic Film Use per Unit Grain Sown Area (kilograms per hectare)	B4	-
		Agricultural Water Use per Unit Grain Sown Area (ten thousand cubic meters per hectare)	B5	-
	Recoverability of Grain Production	Affected/Damaged Area (%)	B6	-
		Soil Erosion Control Area (thousand hectares)	B7	+
		Multiple Cropping Index (%)	B8	+
		Increase in Agricultural Gross Output Value (billion yuan)	B9	+
Transformative capacity	Diversity and Collaboration in Grain Productio	Crop Diversification (%)	C1	+
		Feed Grain Sown Area as a Proportion of Grain Sown Area (%)	C2	+
		Agriculture,Forestry,Animal Husbandry, and Fishery Service Output Value/Total Agricultural Output Value (%)	C3	+
	Innovation in Grain Production	Fiscal Expenditure on Agriculture, Forestry, and Water Affairs (billion yuan)	C4	+
		Agricultural Research Expenditure (billion yuan)	C5	+
		Personnel Engaged in Agricultural Scientific Activities (ten thousand people)	C6	+

Note: The weighting coefficients are as follows: coefficient a = grain sown area/total crop sown area, coefficient b = agricultural output value/total agricultural, forestry, animal husbandry, and fishery output value. The indicators A1, B1, B2, B3, B4, B5, B7, C5, and C6 are each multiplied by coefficient a; the indicators A3, B9, and C4 are each multiplied by coefficient a × coefficient b.

Resistance capacity in food production refers to a system’s capacity to withstand unpredictable shocks, serving as a measure of its overall functional robustness. This study conceptualizes resistance capacity through two primary dimensions: foundational security of food production and stability of production capacity. Foundational security encompasses the essential conditions that underpin food production, providing a basis for assessing resource input levels. Specifically, this study utilizes three tertiary indicators to capture this dimension: effective irrigated area, grain sown area, and workforce size within primary industries. Stability of production capacity, on the other hand, reflects the consistency of food production outputs. A higher level of output stability indicates enhanced system resistance, which is assessed here via metrics such as the coefficient of grain yield fluctuation, per capita grain availability, and grain yield per unit sown area—each reflecting the scale and stability of production capacity.

Restorative capacity denotes the system’s ability to recover to normal production levels following exposure to external shocks. This study examines restorative capacity across two secondary dimensions: ecological coordination and recoverability. Ecological coordination assesses the degree of alignment between food production processes and environmental systems; a stronger alignment reduces the rigidity of resource and environmental constraints, promoting sustainability. To quantify this coordination, this study applies negative indicators, including pesticide usage per unit sown area, agricultural diesel consumption, fertilizer application rates, agricultural plastic film use, and agricultural water consumption. These indicators offer insight into the ecological demands of food production and its sustainability profile. Recoverability in food production indicates the system’s capability to resume normal production levels following external shocks. This ability is assessed by examining the impact of disasters, ecological protection and restoration efforts, and other contributing factors. Specifically, metrics such as disaster/damage area, soil erosion control area, and crop rotation index are used to gauge the system’s resilience. Additionally, economic growth plays a pivotal role in enabling food production systems to recover from external shocks, representing the underlying vitality of regional food production. This is quantified by the added value of total agricultural output, which serves as a proxy for regional economic growth and its capacity to support recovery.

Transformative capacity in food production refers to the system’s ability to not only withstand unpredictable shocks but also to forge new growth pathways in response. This study conceptualizes transformative capacity through two secondary indicators: diversity and collaboration in food production and innovation in food production. Food production diversity encompasses metrics such as crop diversification, the proportion of feed grain sown area relative to the total grain sown area, and the ratio of output from agricultural services to the overall output in agriculture, forestry, animal husbandry, and fishery sectors. Here, crop diversification is defined as the proportion of non-grain crop sown area to the total crop sown area, reflecting the breadth and adaptability of production. Innovation in food production represents the role of funding and technological support in enhancing the quality and efficiency of production activities. This study includes four primary indicators to measure this dimension: fiscal expenditure on agricultural, forestry, and water affairs; agricultural research expenditure; and the workforce in agricultural science and technology. The latter two indicators—agricultural research expenditure and agricultural science personnel are calculated as follows: Agricultural research expenditure = total research and development expenditure × (agricultural output/total output of agriculture, forestry, animal husbandry, and fishery). Agricultural science and technology personnel =total number of science and technology workers × (agricultural output/total output of agriculture, forestry, animal husbandry, and fishery). Together, these metrics provide a robust depiction of the system’s innovative capacity and its ability to achieve higher production quality and resilience through strategic investments in financial and technological resources.

#### 3.2.2 Explanatory variable

The core explanatory variable in this study is the agricultural land “three rights separation” policy. Building on the work of Cao ZJ et al. [16]. and Gong MG and Chen SF [17], the commencement of each province’s comprehensive land rights certification and registration marks the initiation of the policy. This approach generates a binary dummy variable for the “three rights separation” policy, coded as 1 for years following the reform’s implementation and 0 for years preceding it. Table 2 provides the sequence and timeline for land certification implementation across the 30 provinces studied.

**Table 2:** Start time of confirmation of agricultural land rights and registration in each province.

Start Time	Provinces
2014	Anhui, Sichuan, Shandong
2015	Jiangsu, Jiangxi, Hubei, Hunan, Gansu, Ningxia, Jilin, Guizhou, Henan, Shanghai
2016	Hebei, Shanxi, Inner Mongolia, Liaoning, Heilongjiang, Zhejiang, Guangdong, Hainan, Yunnan, Shaanxi
2017	Beijing, Tianjin, Fujian, Guangxi, Qinghai, Chongqing
2018	Xinjiang

#### 3.2.3 Mechanism variables

To verify the mechanisms by which the separation of three rights of agricultural land impacts the resilience of grain production in China, this study constructs variables for land transfer and productive agricultural investment to test the theoretical hypotheses. Drawing from relevant literature and considering data availability, land transfer is measured by the ratio of transferred land area to household contracted land area [33]; productive agricultural investment is measured by the area harvested by machinery, with the area subject to coefficient a stripping.

#### 3.2.4 Control variables

Apart from the impact of the separation of three rights of agricultural land on grain production resilience, other factors also influence this resilience, and thus, need to be controlled within the model. The following control variables have been selected for this study: regional economic development level, measured by per capita GDP and logarithmically transformed for normalization; urbanization rate, measured by the proportion of the urban population to the total population; rural human capital, measured by the average years of education among rural residents; farmer income level, measured by the per capita disposable income of rural residents; agricultural industry agglomeration, measured using location entropy to assess the level of industry concentration(The calculation method for agricultural industry agglomeration is in Appendix A.) ; industrial structure, measured by the proportion of the primary industry’s output to GDP; and agricultural insurance density, measured by the ratio of agricultural insurance premiums to the number of individuals employed in the primary industry.

### 3.3 Data sources

Considering data availability and completeness, this study includes 30 provinces in China (excluding Tibet, Hong Kong, Macau, and Taiwan) as the research sample, covering the period from 2005 to 2021. The research data primarily come from the “China Statistical Yearbook,” “China Rural and Agricultural Statistics Yearbook,” “China Science and Technology Statistics Yearbook,” “China Water Resources Yearbook,” “China Rural Operations Management Annual Report,” as well as the National Bureau of Statistics, provincial statistical yearbooks, the EPS database, and the Breck Agricultural Database platform. Missing data were filled using linear interpolation. Descriptive statistics for the main variables are shown in Table 3.

**Table 3:** Descriptive statistics of major variables.

Type	Variable Symbol	Variable Name	Unit	Mean	Standard Deviation	Minimum	Maximum
Dependent Variable	Resil	Resilience Level of Grain Production	—	0.2447	0.0964	0.0865	0.5431
Core Explanatory Variable	DID	The Rights Separation	—	0.3686	0.4829	0.0000	1.0000
Control Variables	Lnrgdp	Regional Economic Development Level	Yuan	10.5002	0.6580	8.9958	11.9940
	Urban	Urbanization Rate	%	0.5579	0.1396	0.2989	0.8930
	Edu	Rural Human Capital	—	7.5756	0.6638	5.8476	9.4089
	Income	Farmers’ Income Level	Yuan	1.0282	0.6383	0.2134	3.4754
	Agg	Agricultural Industrial Agglomeration	—	1.0186	0.4171	0.0918	1.8500
	Struc	Regional Industrial Structure	—	0.1064	0.0563	0.0028	0.2584
	Insure	Agricultural Insurance Density	million per person	261.3735	431.1287	0.0217	3049.6670
	Transfer	Farmland Transfer Rate	%	0.2413	0.1764	0.0136	0.7480
	Invest	Mechanized Farming Area	Thousand hectares	1771.41	2391.1720	18.8131	14059.17

## 4 Empirical results and analysis

### 4.1 Measurement and analysis of grain production resilience levels

Based on the grain production resilience evaluation indicator system constructed from Table 1, this paper calculates the resilience levels of grain production in 30 provinces in China from 2005 to 2021 as shown in Table 4. Due to space limitations, only the years 2005, 2009, 2013, 2017, and 2021 are listed here.

**Table 4:** Comprehensive scores of China’s grain production resilience.

Provinces	2005	2009	2013	2017	2021	2005–2021 Rate of Change (%)
Beijing	0.1494	0.1765	0.1931	0.1995	0.2394	160.21
Tianjin	0.1247	0.1341	0.1556	0.1633	0.1773	142.23
Hebei	0.2702	0.3061	0.3498	0.3882	0.4127	152.71
Shanxi	0.1956	0.2197	0.2544	0.2727	0.2866	146.52
Inner Mongolia	0.2299	0.2683	0.3154	0.3538	0.3998	173.92
Liaoning	0.2180	0.2314	0.2619	0.2899	0.3087	141.59
Jilin	0.2244	0.2427	0.2649	0.2920	0.3040	135.46
Heilongjiang	0.2547	0.3206	0.4128	0.4832	0.5337	209.51
Shanghai	0.0998	0.1203	0.1415	0.1549	0.1936	194.04
Jiangsu	0.2206	0.2863	0.3772	0.4539	0.5439	246.52
Zhejiang	0.1413	0.1571	0.1960	0.2116	0.2607	184.50
Anhui	0.2226	0.2559	0.2968	0.3510	0.3875	174.08
Fujian	0.1211	0.1360	0.1589	0.1528	0.1747	144.30
Jiangxi	0.1631	0.1878	0.2033	0.2319	0.2593	158.92
Shandong	0.2791	0.3436	0.4059	0.4820	0.5432	194.59
Henan	0.3155	0.3625	0.3939	0.4447	0.5110	161.98
Hubei	0.1799	0.2084	0.2517	0.3017	0.3327	184.90
Hunan	0.2033	0.2373	0.2706	0.2978	0.3104	152.66
Guangdong	0.1738	0.2109	0.2665	0.2882	0.3751	215.79
Guangxi	0.1636	0.1778	0.1926	0.2078	0.2277	139.18
Hainan	0.0787	0.0878	0.0944	0.0910	0.1077	136.84
Chongqing	0.1558	0.1631	0.1793	0.1972	0.2248	144.23
Sichuan	0.2340	0.2726	0.3202	0.3850	0.4343	185.55
Guizhou	0.1625	0.1870	0.2078	0.2367	0.2483	152.82
Yunnan	0.2020	0.2173	0.2502	0.2703	0.3123	154.59
Shanxi	0.2256	0.2472	0.2666	0.2986	0.3371	149.40
Gansu	0.1998	0.2237	0.2487	0.2624	0.2787	139.49
Qinghai	0.0895	0.0882	0.0939	0.0970	0.1010	112.83
Ningxia	0.1151	0.1338	0.1444	0.1576	0.1668	144.85
Xinjiang	0.1608	0.1745	0.2127	0.2264	0.2519	156.60
Major Grain Producing Areas	0.2320	0.2710	0.3173	0.3658	0.4062	175.13
Major Grain Marketing Areas	0.1270	0.1461	0.1723	0.1802	0.2184	171.98
Balanced Production and Marketing Areas	0.1670	0.1832	0.2051	0.2227	0.2435	145.78
National Average Level	0.1858	0.2126	0.2460	0.2748	0.3082	165.83

Temporally,during the observation period,the resilience level of grain production in China generally showed an upward trend,with the national average increasing from 0.1858 in 2005 to 0.3082 in 2021, a growth of nearly 1.66 times. However,the overall resilience level of grain production remains not high,with significant room for growth. Since the new century, to ensure national food security, China has abolished the agricultural tax and has been continuously increasing subsidies for grain production, which has enhanced farmers’ enthusiasm for grain cultivation. Since the 18th National Congress, food security has been elevated to a national security strategy, and strategies such as storing grain in the land and technologies,as well as initiatives like high-standard farmland construction,enhancement of agricultural mechanization, and revitalization of the seed industry,have been implemented,which have contributed to the sustained improvement of grain production resilience. Nevertheless, China’s grain production still faces multiple challenges such as resource and environmental constraints, insufficient development of agricultural technology, inadequate farmer enthusiasm for grain cultivation, and extreme climate changes, making the path to enhancing grain production resilience arduous and long.

**Fig 1:**
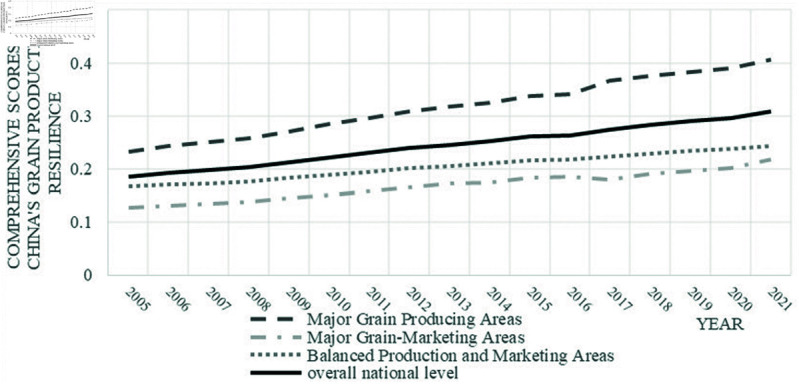
2005–2021 Development trends in grain production resilience in China’s three major functional zones.

From the perspective of the three main functional zones for grain production , as illustrated in Fig 1, the resilience levels of these zones followed a general upward trend during the observation period, mirroring the national trajectory, though significant disparities were observed between zones. Overall, the distribution pattern was as follows: major production areas > balanced production and marketing areas > major marketing areas. The resilience of grain production in major production areas consistently surpassed that of both major marketing areas and balanced production and marketing areas, as well as the national average. Furthermore, the resilience gap between major production areas and the other zones has continued to widen, largely due to the richer grain production resources and the preferential allocation of policies and technologies to these areas, aligned with their designated function. While the resilience of grain production in balanced production and marketing areas exceeds that of major marketing areas, it remains below the national average.

The results shown in Table 4 indicate that in 2021, Jiangsu Province led the nation in grain production resilience, achieving a level of 0.5439. Shandong, Heilongjiang, and Henan followed closely, each surpassing a resilience level of 0.5, with only marginal differences among them. On the opposite end, Qinghai Province recorded the lowest resilience at just 0.1010, while Hainan and Ningxia ranked second and third lowest, with scores of 0.1077 and 0.1688, respectively. Additionally, Tianjin, Shanghai, and Fujian also reported resilience scores below 0.2, highlighting significant potential for improvement. Compared to 2005, all provinces showed growth in resilience, with Jiangsu experiencing the most substantial increase at 246.52%,while Qinghai saw the smallest increase at 112.83%. Provinces with resilience growth rates exceeding 200% included Jiangsu, Heilongjiang, and Guangdong. Fifteen provinces exhibited growth rates be-tween 150% and 200%, while the remaining 12 had rates below 150%. These results illustrate that, while the resilience of grain production in China has improved rapidly from 2005 to 2021, considerable disparities between provinces persist.

### 4.2 Baseline regression results

Table 5 presents the estimated effects of the separation of three rights of agricultural land on grain production resilience. The findings show that, after controlling for fixed individual and time effects, the separation of three rights of agricultural land positively impacted grain production resilience, significant at the 1% level. Specifically, for every 1% increase in the effectiveness of the separation of three rights of agricultural land, grain production resilience improved by 7.9%. Overall, the implementation of these policies has had a favorable effect on enhancing grain production resilience, thereby confirming Hypothesis H1. This suggests that future policy efforts should further explore the separation of three rights of agricultural land reforms to provide stronger institutional support for improving grain production resilience.

**Table 5:** Baseline regression model results.

variable	(1)
**DID**	0.0790***
	(0.0295)
**Lnrgdp**	-0.356**
	(0.139)
**Urban**	0.478***
	(0.139)
**Edu**	-0.145***
	(0.0462)
**Income**	0.450***
	(0.102)
**Agg**	0.154**
	(0.0640)
**Struc**	0.208***
	(0.0656)
**Insure**	-0.159***
	(0.0328)
**Constant**	3.704**
	(1.465)
**Time fixed**	Yes
**Individual fixed**	Yes
**Observations**	510
**R-squared**	0.954

Note: Robust standard errors in parentheses,*** p<0.01, ** p<0.05, * p<0.1.

### 4.3 Parallel trends test

The reliability of the DID model estimation results hinges on passing the parallel trends test, which requires no significant differences in regression coefficients in the years preceding the implementation of the “three rights separation” policy, while significant differences should emerge post-implementation. This study applies the event study method proposed by Jackson L S et al. [34]. and employs Formula (2) to conduct the test. To mitigate multicollinearity and potential interference from other policies, this paper follows the methodology of Xu W and Zhang JH [35], shortening the sample period and using panel data from 2010 to 2018 for the parallel trends test, with 2013 as the baseline group.

**Fig 2:**
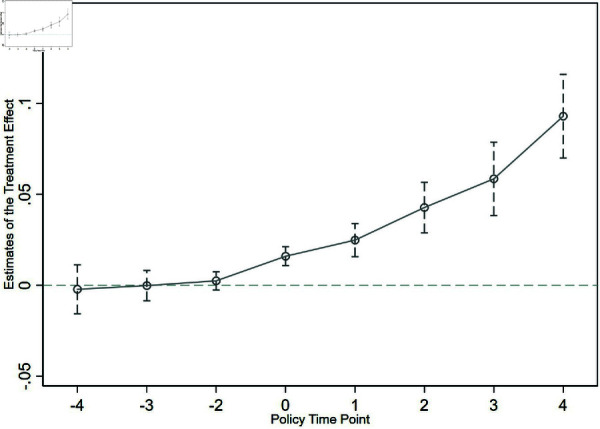
Parallel trend test dynamic effect plot.

In Fig 2, the changing trend of the regression coefficient $\beta_{t}$ is illustrated. The corresponding estimated parameters are represented by round dots, while the 95% confidence intervals are indicated by the vertical lines. On the horizontal axis, the value –4 represents four years prior to the policy’s implementation, 0 indicates the initial year of implementation (2014), and 4 represents the fourth year after implementation. The results reveal that, prior to policy implementation, the estimated policy coefficients remain close to zero, showing no significant differences in the interaction term estimates before 2014. Post-implementation, the confidence intervals for these estimates consistently exceed zero, indicating significant differences. These findings confirm that the parallel trends test has been successfully passed, affirming the reliability of the baseline regression results and demonstrating that the implementation of the agricultural land “three rights separation” policy significantly enhances grain production resilience.

### 4.4 Robustness tests

#### 4.4.1 Placebo test

**Fig 3:**
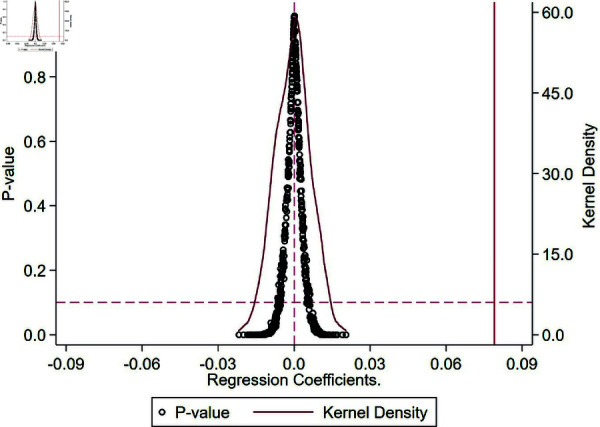
Placebo test plot.

To ensure that the baseline regression results are not influenced by other policies or unobservable random factors, a double-random counterfactual estimation framework was constructed based on the timing and location of the separation of three rights of agricultural land implementation. This test was used to assess the authenticity of the effects generated by the randomly assigned the separation of three rights of agricultural land. As shown in Fig 3, following 500 randomly generated policy shocks, the simulated estimation coefficients for grain production resilience were found to follow a normal distribution with a mean close to 0. The actual estimated coefficient of 0.073, indicated by the vertical dashed line, stands out as an outlier within the distribution of simulated coefficients. This finding suggests that the positive effect of the separation of three rights of agricultural land on grain production resilience is not a random occurrence, thereby affirming the robustness of the study’s conclusions.

#### 4.4.2 Substitution of the dependent variable

Grain total output, as a critical indicator of both grain production resilience and food security, was used to substitute the dependent variable in Formula (1) for a robustness check, employing the variable substitution method. As shown in column (1) of Table 6, the regression coefficient remains significantly positive, further confirming the robustness of the model.

**Table 6:** Robustness tests.

variable	(1)	(2)	(3)	(4)	(5)
	Replace Dependent Variable	Lag Core Explanatory Variable by One Period	Adjust Sample Size: Exclude 2005	Adjust Sample Size: Include 2022	Exclude Beijing, Hebei, and Shanxi
**DID**	0.054**	0.114***	0.056**	0.059**	0.083***
	(0.0254)	(0.0338)	(0.0276)	(0.0284)	(0.0311)
**Cons_**	0.656	3.866***	3.851***	4.190***	4.793**
	(1.3270)	(1.4815)	(1.4862)	(1.5306)	(1.9176)
**Control variable**	Yes	Yes	Yes	Yes	Yes
**Time fixed**	Yes	Yes	Yes	Yes	Yes
**Individual fixed**	Yes	Yes	Yes	Yes	Yes
**Observations**	510	480	480	540	459
**R-squared**	0.966	0.954	0.959	0.952	0.952

Note: Robust standard errors in parentheses,*** p < 0.01, ** p < 0.05, * p < 0.1.

#### 4.4.3 Lagging the core independent variable by one period

Given that the separation of three rights of agricultural land may not only impact grain production resilience in the current year but may also have long-term effects, the core independent variable was lagged by one period to conduct a robustness test. The estimation results, presented in column (2) of Table 6, show that the regression coefficient for the separation of three rights of agricultural land on grain production resilience remains significantly positive at the 1% level, thereby passing the robustness test.

#### 4.4.4 Adjusting the sample size

First, the sample size was adjusted by time. After excluding 2005 and including 2022, the regression estimation was re-conducted. As shown in columns (3) and (4) of Table 6, the coefficient results remain significant. Second, the sample size was adjusted by individual regions. The provinces ranked first in the administrative code order in the major grain-producing areas, major marketing areas, and balanced production and marketing areas, namely Hebei, Beijing, and Shanxi, were removed. The empirical results in column (5) of Table 6 remain consistent with the baseline regression results. Thus, regardless of how the sample size is adjusted, the implementation of the separation of three rights of agricultural land has significantly improved grain production resilience.

## 5 Further analysis

### 5.1 Mechanism analysis

The empirical results indicate that a significant positive impact on improving grain production resilience has been caused by the separation of three rights of agricultural land. It is suggested by theoretical analysis that this effect occurs through a transmission mechanism, primarily by facilitating land transfers and increasing agricultural productive investments. In the following section, the internal mechanisms by which the separation of three rights of agricultural land enhances grain production resilience will be further investigated.

To test these mechanisms, a two-step approach has been adopted in this paper, following the method outlined by Jiang T [36] to analyze the mediating effect. In the first step, the core independent variable is regressed on the dependent variable, as shown in column (1) of Table 5. In the second step, the core independent variable is regressed on the mediating variable. If significance is found in the second regression, the mediating effect is confirmed, supported by the theoretical analysis of the mechanism. The results of this mechanism test are presented in Table 7.

**Table 7:** Mechanism analysis.

variable	(1)	(2)
	Transfer	Invest
**DID**	0.175*	0.098**
	(0.0718)	(0.0463)
**Cons_**	3.538	2.804
	(2.219)	(2.088)
**Control variable**	Yes	Yes
**Time fixed**	Yes	Yes
**Individual fixed**	Yes	Yes
**Observations**	510	510
**R-squared**	0.912	0.930

Note: Robust standard errors in parentheses,*** p < 0.01, ** p < 0.05, * p < 0.1.

To examine the mechanism related to land transfer, this paper uses the proportion of land transferred in a given province relative to the total contracted land area in that year to quantify the level of land transfer. By substituting this variable into the baseline regression model (1), the results, shown in column (1) of Table 7, reveal a coefficient of 0.175 for the effect of the separation of three rights of agricultural land on land transfer, significant at the 1% level. This indicates that the policy’s implementation has substantially promoted land transfer. Motivated by land transfer incentives, farmers with low willingness or profitability in grain farming activities transfer the management rights of their farmland to larger-scale production and operation entities. This ensures that the overall grain sowing area does not decline, improves land use efficiency and output efficiency, and further enhances the resilience of grain production in the face of external shocks. Land transfer fosters specialized division of labor, increasing agricultural production efficiency and risk resistance. Specialized division of labor enables better responses to market fluctuations and natural disasters, thereby strengthening the resilience of the grain production system. After land transfer, operators have greater motivation and resources for technological innovation, such as introducing efficient planting techniques and pest and disease control methods, to improve the quality and yield of grain production. Additionally, land transfer promotes innovation in management, such as through strategic planting plans and efficient supply chain management, thereby enhancing the transformative capacity of the grain production system. Furthermore, the large-scale operators formed after land transfer can more easily access financial instruments like green credit and green insurance, providing sustainable financial support for grain production. This addresses financing challenges in the grain production process, reduces risks associated with grain production and operation, and strengthens the resilience of grain production.

In investigating the mechanism of agricultural productive investment, this study utilizes the grain machine-harvested area in a given year from a specific province as a proxy for grain productive investment . By substituting this variable for the dependent variable in the baseline regression model (1), a re-estimation is conducted. The results, presented in column (2) of Table 7, show a significantly positive coefficient for the effect of the separation of three rights of agricultural land on grain productive investment. This finding suggests that the implementation of the policy substantially stimulates investment in grain production. Agricultural productive investments can be utilized to purchase advanced agricultural technologies and equipment, such as mechanized farming equipment, drone-based pest control, smart irrigation systems, and precision agriculture technologies. These technologies enhance the stability and disaster resilience of grain production. Additionally, agricultural productive investments can be directed towards the research, development, and promotion of new grain production techniques, thereby improving grain production efficiency and quality. For instance, biotechnology can cultivate higher-yielding and more disease-resistant crop varieties, while information technology enables intelligent management of grain production. Moreover, investing in infrastructure for farmland water conservancy, such as constructing irrigation canals, drainage systems, windbreaks, and mechanized farming roads, can effectively mitigate the impact of natural disasters on grain production. Furthermore, increasing investments in farmer education and training enables farmers to master advanced cultivation techniques, management methods, and market information, thus enhancing their management of grain production. On the other hand, when grain production faces external shocks, farmers can respond and make decisions more swiftly and scientifically, thereby enhancing the resilience of grain production.

### 5.2 Heterogeneity analysis

#### 5.2.1 Heterogeneity in natural geographic location

Given the variations in natural resource endowment, geographic location, and levels of economic development, the impact of the separation of three rights of agricultural land on grain production resilience may differ across regions. This study categorizes China’s 30 provinces (including municipalities and autonomous regions) into three regions—eastern, central, and western—to explore the heterogeneity based on geographic location. The results, displayed in columns (1) to (3) of Table 8, indicate that the policy’s effect in the central region is significantly positive, whereas the estimated coefficients for the eastern and western regions are not statistically significant. This suggests that the separation of three rights of agricultural land has notably enhanced grain production resilience in the central region. A possible explanation for this is that the western region, with its less favorable resource endowments for grain production and lower levels of economic development, experiences limited effects of the separation of three rights of agricultural land on agricultural scaling and standardization. In contrast, the eastern region, characterized by a more developed economy and higher land value, tends to prioritize the growth of secondary and tertiary industries over agricultural production. The central region, comprising the largest number of key grain-producing provinces, possesses a stronger agricultural foundation and bears greater political responsibility for ensuring national food security. Consequently, the policy’s impact on promoting land transfers at scale and enhancing agricultural productive investment is more pronounced in this region.

**Table 8:** Heterogeneity analysis.

	(1)	(2)	(3)	(4)	(5)	(6)	(7)	(8)
variable	Eastern	Central	Western	Major Grain Producing Areas	Major Grain Marketing Areas	Balanced Production and Marketing Areas	Southern Rice Planting Region	Northern Wheat Planting Region
**DID**	0.066	0.116*	0.052	0.077*	0.022	0.005	0.071*	0.101**
	(0.0585)	(0.0623)	(0.0410)	(0.0459)	(0.0730)	(0.0342)	(0.0393)	(0.0435)
**Cons_**	2.106	4.698	0.051	-1.994	12.934***	0.661	9.335***	-4.382*
	(4.2724)	(3.5531)	(2.3128)	(2.3522)	(3.1148)	(1.4835)	(3.0486)	(2.5805)
**Control variable**	Yes	Yes	Yes	Yes	Yes	Yes	Yes	Yes
**Time fixed**	Yes	Yes	Yes	Yes	Yes	Yes	Yes	Yes
**Individual fixed**	Yes	Yes	Yes	Yes	Yes	Yes	Yes	Yes
**Observations**	187	136	187	221	119	170	255	238
**R-squared**	0.965	0.977	0.966	0.970	0.961	0.978	0.939	0.959

Note: Robust standard errors in parentheses,*** p < 0.01, ** p < 0.05, * p < 0.1.

#### 5.2.2 Heterogeneity in natural geographic location

Given that different grain production functional zones may influence the impact of the confirmation of agricultural land rights policy, this study classifies the sample into major grain-producing areas, major grain-marketing areas, and balanced production and marketing areas, based on the previously discussed categorization of the three primary grain production functional zones. The regression results, presented in columns (4) to (6) of Table 8, reveal that the policy’s effect in major grain-producing areas is significantly positive, while the estimated coefficients for major marketing areas and balanced production and marketing areas are not statistically significant. The key reason for this disparity lies in the fact that major grain-producing areas constitute the core foundation of national food security. These areas generally benefit from more favorable conditions for grain production, higher levels of factor input, and greater yields compared to major marketing and balanced production and marketing areas. Moreover, the distinctive role of major grain-producing areas makes them more subject to national regulatory oversight and institutional arrangements. As a result, the separation of three rights of agricultural land has a notably stronger effect on enhancing grain production resilience in major grain-producing areas than in major marketing or balanced production and marketing areas.

#### 5.2.3 Heterogeneity in the main grain planting patterns between the North and South

Due to variations in climate and agricultural resource endowment, China has developed a distinctive grain cultivation pattern, divided by the “Qinling Mountains–Huai River” line. In the north, wheat is predominantly grown in drylands, while the south focuses on rice cultivation in paddy fields. The northern regions, located north of the Qinling–Huai River line, mainly engage in wheat cultivation on drylands, whereas the southern regions, south of the line, are characterized by rice cultivation in paddy fields. Given these regional differences, this paper deems it essential to examine the impact of the separation of three rights of agricultural land on grain production resilience in both the northern and southern regions.

The first step in conducting a heterogeneity analysis of the policy’s effects between these two regions is to define the northern and southern grain planting zones. Following the classification method of Geng PP and Luo Bl [37], we categorize Beijing, Tianjin, Hebei, Shanxi, Liaoning, Jilin, Heilongjiang, Shandong, Henan, Shaanxi, Gansu, and Xinjiang as primarily dryland wheat-producing areas. Conversely, Shanghai, Jiangsu, Zhejiang, Anhui, Fujian, Jiangxi, Hubei, Hunan, Guangdong, Guangxi, Hainan, Chongqing, Sichuan, Guizhou, Yunnan, and Tibet are classified as predominantly paddy field rice-producing areas. Since Inner Mongolia, Qinghai, and Ningxia are mainly pastoral regions, they are excluded from the sample in this north-south planting zone analysis. Additionally, considering that provinces like Jiangsu and Anhui straddle the north-south dividing line, this paper follows the method suggested by Luo Bl and Zhang L [38], including both Jiangsu and Anhui in the samples for both the northern and southern regions.

The results of the heterogeneity analysis on the impact of the separation of three rights of agricultural land on grain production resilience between the northern and southern planting zones are presented in columns (7) and (8) of Table 8. The regression coefficients for both regions are significantly positive, with the estimated coefficient for the southern region being 0.071 and for the northern region 0.101. This suggests that the implementation of the separation of three rights of agricultural land has positively contributed to grain production resilience in both northern and southern regions, though the effect is more pronounced in the north than in the south.

## 6 Conclusions, discussion, and policy recommendations

### 6.1 Conclusions

Based on the previous research findings, this paper draws the following conclusions:

(1) From 2005 to 2021, China’s grain production resilience exhibited a steady upward trend, although the overall level remains low, indicating significant potential for further improvement. Notable disparities exist in grain production resilience across the three major functional zones, following the pattern of major grain-producing areas > balanced production and marketing areas > major grain-marketing areas. In 2021, Jiangsu Province ranked first in the nation for grain production resilience, recording the highest growth among all provinces, with an increase of 246.52%.
(2) The separation of three rights of agricultural land has a significantly positive impact on grain production resilience, with notable improvements at the 1% statistical level. The effect of the policy on enhancing grain production resilience is stable and sustainable. After conducting a series of robustness checks,such as substituting the dependent variable and adjusting the sample size—the estimated conclusions remain robust.
(3) The mechanism analysis identifies two pathways through which the separation of three rights of agricultural land enhances grain production resilience. First, the policy promotes land transfers, thereby improving resilience. Second, it encourages farmers to increase their investment in agricultural production, which further strengthens resilience.
(4) The heterogeneity analysis reveals that the policy’s impact on grain production resilience varies according to China’s natural geographical regions, grain production functional zones, and north-south grain planting patterns. Geographically, the policy exerts a significantly positive effect on grain production resilience in the central region, while its impact in the eastern and western regions is not significant. Regarding functional zones, the policy’s influence is more pronounced in major grain-producing areas. As for the north-south grain planting pattern, the policy positively affects grain production resilience in both regions, with a stronger effect observed in the northern region.

### 6.2 Discussion

In the context of food security, this article investigates how the separation of the three rights in agricultural land reform impacts the resilience of grain production. The theoretical analysis identifies two mechanisms through which this reform affects resilience: land transfer and agricultural production investment. Utilizing statistical data from 30 provinces in China from 2005 to 2021 and applying a multi-period difference-in-differences (DID) model, the empirical analysis demonstrates a positive up-ward trend in the resilience of China’s grain production over the years, with the separation of the three rights in agricultural land reform contributing to this increase.

Land, as the primary factor in agriculture, is widely recognized as crucial for enhancing grain production. However, its fixed location and stable area inherently limit its potential contribution. Unlike other inputs, institutional factors do not directly impact grain production and agricultural development; rather, they modulate the effectiveness of inputs such as land, capital, technology, and labor. As the core component of land institutions, the agricultural land property rights system influences not only land inputs but also other production factors in agriculture. Thus, examining the impact of property rights reform on grain production within the constraints of agricultural land resources is of critical importance. Building a resilient grain production system is fundamental to mitigating uncertain risks and safeguarding food security. Research on grain production resilience is still in its early stages, with studies remaining limited, particularly regarding the impact of specific policies on resilience. While scholars have explored the concept of ’three rights’ separation within the context of property rights, few have investigated its effects on enhancing grain production resilience and securing food security. This article addresses this gap by leveraging China’s separation of the three rights in agricultural land reform as a quasi-natural experiment to assess the impact of agricultural land property rights reform on grain production resilience. This study offers several contributions: first, it expands the scope of resilience measurement in grain production; second, it provides both theoretical and empirical insights into the influence of three rights separation in agricultural land reform on resilience; and finally, it presents policy recommendations for fully implementing rural property rights reform to strengthen resilience in grain production.

While this study provides valuable insights, several limitations should be noted. First, due to constraints in data availability, the analysis was limited to the provincial level. Additionally, the complex relationship between the separation of agricultural land rights and grain production resilience involves micro-level stakeholders, such as individual farmers, rural households, and agricultural enterprises, which this study does not encompass. Furthermore, the analysis includes mediation tests only on land transfer and agricultural production investment. Future research could benefit from examining the effects of agricultural land rights separation and grain production resilience at the city or county levels within China. Expanding the research focus to encompass micro-level land operators and incorporating additional variables may also offer a more comprehensive understanding of potential mediation and moderation effects.

### 6.3 Policy recommendations

Based on the research conclusions, this paper offers the following policy recommendations:

(1) Governments at all levels should enhance the promotion of confirmation of agricultural land rights policies, elevate the legal status and value of land rights certificates, and increase their recognition in rural markets. Furthermore, efforts should be made to strengthen the protection of land use and property rights, thereby reinforcing farmers’ sense of security and stability regarding land property rights.
(2) Governments should expedite the establishment of farmland transfer market centers and information exchange platforms, standardize the operational mechanisms of farmland transfer markets, develop clear regulations for land transfers, ensure the enforcement of contracts, and mitigate moral hazards associated with contract execution. Additionally, improving price formation mechanisms for farmland transfers and reducing transaction costs are essential. These efforts will promote the standardization, scaling, and specialization of grain production, ultimately improving production efficiency.
(3) The government should raise awareness among farmers and financial institutions about land contract management rights as collateral. The government should encourage banks at all levels and other financial institutions to explore models of mortgage loans based on agricultural land contracting and management rights, expand financing channels for farmers’ agricultural production, and provide financial support for the expansion of grain reproduction..
(4) The government should explore incentive and subsidy models for different scales of grain-producing households in the areas of purchasing grain cultivation machinery, socialized services for grain production, and the construction of small-scale agricultural water conservancy facilities, in order to provide technical support for the resilience of grain production.
(5) The government should strongly advocate for green grain production. Efforts should be made to increase the adoption rates of soil testing and formula fertilization techniques, green pest control methods, pest monitoring technologies, and water-saving irrigation systems, thereby ensuring the scientific, precise, and efficient use of production inputs. The costs associated with green grain production technologies can be reduced through measures such as financial subsidies and tax incentives. For instance, the prices of organic fertilizers and eco-friendly pesticides could be decreased.
(6) The government must rigorously enforce the arable land protection system. In accordance with the principle of “ensuring no reduction in the quantity, no decline in quality, and no ecological damage to arable land,” the system for safeguarding the quantity, quality, and ecology of arable land should be enhanced. A comprehensive protection assessment system should be established, and the responsibilities of arable land resource supervision departments should be rigorously enforced. The standards for identifying arable land destruction should be clarified, and penalties for destructive behaviors should be increased accordingly. Additionally, innovative models for high-standard farmland construction should be actively explored and promoted, to encourage the participation of agricultural enterprises, cooperatives, family farms, and large-scale grain producers in such projects. Furthermore, regions should be encouraged to develop land reclamation and re-cultivation models that are tailored to local conditions.

## Supporting information

S1 Data The data used in this article is provided in S1 Data. (XLSX)
